# Supplementary material for: Development of chemokine network inhibitors using combinatorial saturation mutagenesis
Source: Commun Biol. 2025 Apr 3;8:549. doi: 10.1038/s42003-025-07778-6 (PMC11969024; doi:10.1038/s42003-025-07778-6)
Supplement: Supplementary file 4 — Supplementary Data 2 [file 42003_2025_7778_MOESM4_ESM.zip › SupplementaryData3/scripts/xcomb.tar!/./script/analysis/JK/SuppTablePeptides.docx]

Supplementary Table X: Peptide Sequences

| Peptide | Sequence | Expected Monomeric Mass Da | Observed Mass Da | Water | PBS | DMSO | HCOOH |
| --- | --- | --- | --- | --- | --- | --- | --- |
| HD2 | EEDDYTAYAPLTCYFT | 1901.02 | 1901.4 | Insoluble | Insoluble | Soluble | NA |
| HD2SCR | TLETDTFYECPDAYAY | 1901.02 | 1902.2 | Insoluble | Insoluble | Soluble | NA |
| HD2E1D | DEDDYTAYAPLTCYFT |  |  |  |  |  |  |
| HD2E2D | EDDDYTAYAPLTCYFT |  |  |  |  |  |  |
| HD2E2W | EWDDYTAYAPLTCYFT |  |  |  |  |  |  |
| HD2Y5W | EEDDWTAYAPLTCYFT | 1924.06 | 1923.6 | Insoluble | Insoluble | Soluble | NA |
| HD2T6D | EEDDYDAYAPLTCYFT | 1915 | 1914.6 | Insoluble | Insoluble | Soluble | NA |
| HD2T6W | EEDDYWAYAPLTCYFT | 1986.13 | 1986.5 | Insoluble | Insoluble | Soluble | NA |
| HD2A7D | EEDDYTDYAPLTCYFT |  |  |  |  |  |  |
| HD2Y8W | EEDDYTAWAPLTCYFT |  |  |  |  |  |  |
| HD2A9W | EEDDYTAYWPLTCYFT | 2016.15 | 2016.2 | Insoluble | Insoluble | Insoluble | Soluble |
| HD2P10D | EEDDYTAYADLTCYFT |  |  |  |  |  |  |
| HD2L11I | EEDDYTAYAPITCYFT | 1901.02 | 1900.8 | Insoluble | Insoluble | Soluble | NA |
| HD2T12I | EEDDYTAYAPLICYFT |  |  |  |  |  |  |
| HD2T12V | EEDDYTAYAPLVCYFT |  |  |  |  |  |  |
| HD2T12W | EEDDYTAYAPLWCYFT | 1987.11 | 1987 | Insoluble | Insoluble | Soluble | NA |
| HD2T16C | EEDDYTAYAPLTCYFC | 1903.06 | 1902.6 | Insoluble | Insoluble | Soluble | NA |
| HD2T16D | EEDDYTAYAPLTCYFD |  |  |  |  |  |  |
| HD2CM1598 | DEDDWTDYADLTCYFC |  |  |  |  |  |  |
| HD2CM2132 | EEDDYWDWADITCYFC |  |  |  |  |  |  |
| HD2CM3085 | DWDDWTAYWDLVCYFD | 2112.24 | 2112.2 | Insoluble | Insoluble | Insoluble | Soluble |
| HD2CM1516 | DWDDYWAYAPLVCYFC |  |  |  |  |  |  |
| HD2CM418 | DDDDYWAYAPLWCYFD |  |  |  |  |  |  |
| HD2CM307 | DEDDWDDYAPITCYFT | 1968.02 | 1967.8 | Insoluble | Insoluble | Insoluble | Soluble |
| HD2CM539 | DDDDWDDYAPITCYFD | 1967.98 | 1967.8 | Insoluble | Insoluble | Insoluble | Soluble |
| HD2CM462 | DDDDWDAYAPITCYFD |  |  |  |  |  |  |
| HD2CM470 | EDDDWDDYAPITCYFD |  |  |  |  |  |  |
| HD2CM325 | EEDDWDDYAPITCYFD |  |  |  |  |  |  |
| HD2CM304 | DEDDWDAYAPIWCYFT | 2009.12 | 2008.8 | Insoluble | Insoluble | Soluble | NA |
| HD2CM155 | EEDDWDAYAPIWCYFT |  |  |  |  |  |  |
| HD2CM452 | DDDDWDAYAPIWCYFT | 1995.09 | 1994.4 | Insoluble | Insoluble | Soluble | NA |
| HD2CM320 | EDDDWDAYAPIWCYFT |  |  |  |  |  |  |
| HD2CM322 | EDDDYDAYAPIWCYFD |  |  |  |  |  |  |
| HD2CM423 | DDDDYWDYAPLTCYFD |  |  |  |  |  |  |
| HD2CM526 | DDDDWWDYAPLTCYFD | 2039.1 | 2039.2 | Insoluble | Insoluble | Soluble | NA |
| HD2CM1578 | EWDDWTDYAPLVCYFD |  |  |  |  |  |  |
| HD2CM1400 | DWDDYWDYAPLTCYFD |  |  |  |  |  |  |
| HD2CM1530 | EEDDWTDYWPITCYFD |  |  |  |  |  |  |
